# Generation of Gait Events with a FSR Based Cane Handle

**DOI:** 10.3390/s21165632

**Published:** 2021-08-21

**Authors:** Andrés Trujillo-León, Arturo de Guzmán-Manzano, Ramiro Velázquez, Fernando Vidal-Verdú

**Affiliations:** 1 Departamento de Electrónica, Universidad de Málaga, 29071 Málaga, Spain; arturodgm94@gmail.com; 2 Instituto de Investigación Biomédica de Málaga (IBIMA), 29071 Málaga, Spain; 3 Facultad de Ingeniería, Universidad Panamericana, Aguascalientes 29020, Mexico; rvelazquez@up.edu.mx

**Keywords:** gait, gait events, gait monitoring, FSR sensors, instrumented cane, assistive technology, prosthesis, exoskeletons

## Abstract

Gait analysis has many applications, and specifically can improve the control of prosthesis, exoskeletons, or Functional Electrical Stimulation systems. The use of canes is common to complement the assistance in these cases, and the synergy between upper and lower limbs can be exploited to obtain information about the gait. This is interesting especially in the case of unilateral assistance, for instance in the case of one side lower limb exoskeletons. If the cane is instrumented, it can hold sensors that otherwise should be attached to the body of the impaired user. This can ease the use of the assistive system in daily life as well as its acceptance. Moreover, Force Sensing Resistors (FSRs) are common in gait phase detection systems, and force sensors are also common in user intention detection. Therefore, a cane that incorporates FSRs on the handle can take advantage from the direct interface with the human and provide valuable information to implement real-time control. This is done in this paper, and the results confirm that many events are detected from variables derived from the readings of the FSRs that provide rich information about gait. However, a large inter-subject variability points to the need of tailored control systems.

## 1. Introduction

Human gait can provide key information about health of the subject under observation. It is related to neurodegenerative diseases such as dementia and others such as osteoporosis. Moreover, it can be used to prevent falls of elderly people. It is also an interesting tool to monitor the progress of patients in rehabilitation processes, for instance those caused by a stroke, or to develop assistance for people with Parkinson, as well as for diagnosis, for instance of persons with paretic limbs. Gait analysis is also exploited in the control of prosthesis and exoskeletons for lower limbs [1,2].

Many of the above mentioned applications require the use of wearable devices for the purpose of gait feature extraction. The most popular are IMUs (Inertial Measurement Units) that are attached to different parts of the lower limbs such as the thigh, shank or foot. The orientation of the segment that is tracked by the device or the angular speed and acceleration provided by the gyroscope and accelerometer of the IMU are typical inputs to this task. Some works report results that show success gait phase detection with only one IMU attached to a segment of each lower limb [3]. Gait phases are time intervals inside the gait cycle or stride that are separated by characteristic events or transitions.

On the other hand, instrumented canes are common assistive devices for old people. They are a non-invasive means to monitor the gait. This benefits vulnerable people such as the elderly or physically or mentally handicapped because the sensors do not have to be attached to certain positions on their bodies but they are integrated in a device they are familiar with. This also improves the acceptance of the assistive device since it eases donning and doffing and even allows walking barefoot [4,5]. Several instrumented canes have been developed to gather different information related to gait. They are devoted to identify different walking activities [6], to assess the adequate use of the cane as walking aid [7,8,9], to detect walking abnormalities and different terrains [10] or to detect risk of falling [11]. Other instrumented canes are used for studies of walking dynamics and gait segmentation [4,12,13,14].

Special attention can be paid to canes as aids in the control of lower limb prosthesis or exoskeletons as well as gait involving Functional Electrical Stimulation (FES). Synchronization of these assistive devices with gait is mandatory to avoid damage to the user, for instance caused by falls, and achieve an acceptable walking performance. IMUs, Force Sensing Resistors (FSR) placed on the feet, encoders or current sensors to obtain information about the motors load are used for this purpose [1,2,15,16]. Nevertheless, some exoskeletons are not able to maintain the user in standing posture [17]. Moreover, soft exoskeletons are intended to assist people that retain some walking ability such as those that have suffered from stroke [18] or children with Cerebral Palsy that can walk with aids [19]. In these cases, canes or crutches are commonly used as a complementary tool. In addition, the currently available exoskeletons usually adapt a pre-programmed gait and so the patient follows the exoskeleton lead. However, synergy between upper and lower limbs motion can be exploited to develop better control algorithms to improve stability [20] or continuous gait performance in continuous walking [21]. The latter reported a strategy for cases where gait is impaired on one side of the body such as in hemiplegic people, the amputee, or those that suffered from stroke [3], and crutches or canes are also needed. The sensor readings from the unaffected leg of an exoskeleton can be used to control the side of the affected leg as reported in [22]. In these cases, since only one side is affected, a unilateral aid can be used, such as a single leg exoskeleton. The synergy between upper and lower limbs movement can be exploited to use an instrumented cane in the unaffected side for the control of the single leg exoskeleton. Results from this strategy are reported in [23] for patients with hemiparesis, where improvements in the symmetry between right and left leg steps and cycle-to-cycle consistency are observed. The angles and angular velocities of the hip and knee joints of the unaffected leg as well as the cane in the sagittal plane are the input to the trajectory planner obtained from the synergies observed in healthy people. Moreover, FSRs in the insole and in the tip of the cane are used to detect the standing, start, stop and continue conditions. In addition, the FSRs in the insole allows detecting the stance and swing phases to apply different gains of the proportional-differential controllers. These gains have previously tuned manually. Eight FSRs in the insole of each shoe are also used in [24] to detect the swing and stance phases together with a gyroscope and an FSR in a glove per hand to recognize walking intention. The FSR sensor for each palm recognizes the on/off status of the crutch.

As commented above, placing the sensors in the insole, gloves, or strapped in some parts of the body can reduce the acceptance of the aid and complicate its usage. An alternative is placing the FSRs on the handle of the cane. This is done in [6], where the output of seven FSRs on the handle is aggregated to that from an FSR at the tip of the handle to obtain two derived variables, the summation of all outputs and the magnitude of a force vector formed by the eight FSR outputs as components together with other gathered sensor data to classify different activities. The same authors use the aggregation of the forces registered by eight FSRs on the handle as an estimation of the grip pressure in a study to assess the cane usage as well as the risk of falls. Eighteen FSRs were mounted also on the handle of a cane in the work reported by the authors in [25]. A good correlation was found between variables derived from these sensors and other sensors in the cane. Specifically, the center of pressure computed with the FSRs in the top side of the handle correlated well with the angle obtained by the IMU in the cane, while the mean of the same readings correlated with the output of a load cell in the shaft. Moreover, force sensors are commonly used to estimate user intention in human-robot interactions. This is especially suitable if there is a direct sensor-human interface, such as that obtained with the FSRs placed on the handle of a cane. Under this assumption, we hypothesized that the variables derived from the FSRs on the handle could not only correlate but also anticipate the signals from the other sensors. The anticipation of gait events may be critical to achieve timely stimulation in real time control or neuroprostheses or functional electrical stimulation [26] and real-time gait analysis is a key issue to control assistive devices such as prostheses and exoskeletons [3]. The conference paper in [27] confirmed our hypothesis but in a limited experiment where only one subject participates. Here we present results from a broader study where a better prototype was developed, twelve subjects participate and more derived variables are examined. Only data from healthy volunteers are analyzed. This is a limitation of the study but it yet provides interesting information since the aids are aimed to achieve a gait as close as possible to that of healthy people (for instance data from healthy people is used to obtain the trajectory planner in [23]). Timing information of singular events of the data as peaks is examined and quantified with a motion tracking system based on IMUs as reference system used for gait segmentation.

This paper is structured as follows. First, Section 2 describes the custom handle equipped with FSRs to capture data of the cane use, as well as the variables extracted for the study. Then, Section 3 details the instrumented cane elements, the reference system for the gait segmentation and the experimental setup. Section 4 describes the different aspects of the experiment: the group of participants, the protocol and the data processing. Section 5 is dedicated to present and discuss the experiment results. Finally, conclusions of the work are given in Section 6.

## 2. Cane Handle

In this section, the design of the cane handle and the variables extracted from the FSRs attached to the latter are described.

### 2.1. Handle Design

**Figure 1:**
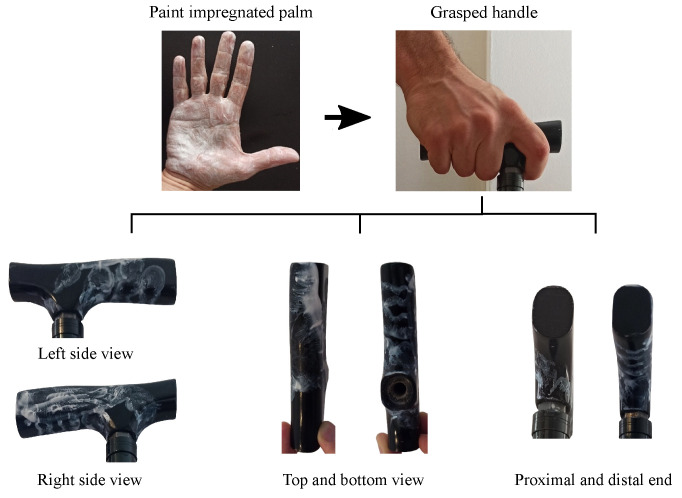
After covering their palm with hand paint, the volunteers grasped the handle. The painted surface helped locate the FSR sensors in an optimal way.

The purpose of this paper is showing the use of a tactile array or a set of FSRs attached on the handle of a cane as a means to detect gait events. A previous step in the design of the handle is to explore where the FSR units should be located. These locations must be those indicated by the areas of contact between the hand palm and the handle while the cane is grasped. A simple experiment was carried out to find out where these contact areas are. Six volunteers (3 males, 3 females; aged between 25 and 79, with a mean of 45.2 and a SD of 20.5 years) covering a range of different shape and hand sizes were invited to grasp the handle of a commercial cane with the palm previously impregnated by hand paint. Figure 1 illustrates the process carried out to obtain the handle surface in contact with the palm for one of the volunteers.

**Figure 2:**
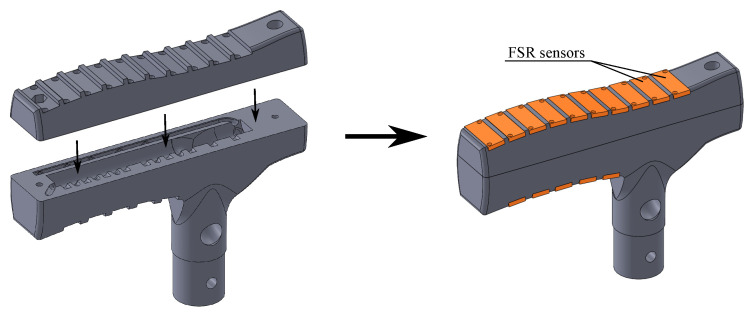
CAD handle design with the FSR sensors in orange.

After the analysis of the obtained images, the distribution of the FSRs was decided and a custom handle was designed and 3D-printed to host them. It consists of an upper and lower part that, when assembled, form a bolted structure with some inner space for the FSR wiring. Figure 2 shows the resulting design, whose shape is based on a conventional model, with the locations of the FSR sensors in orange.

**Figure 3:**
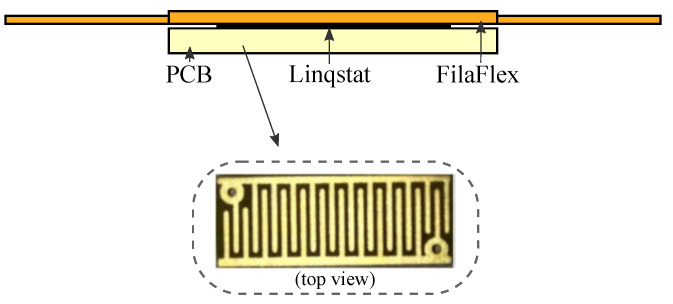
Custom FSR layer structure. Detail of the PCB with the comb electrodes.

**Figure 4:**
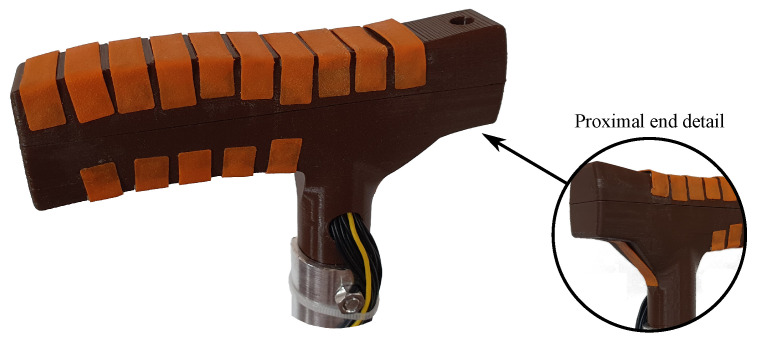
Custom handle implementation.

The FSR sensors are also custom. They are fabricated following a simple approach that consist of placing a sheet of the semiconductive material *Linqstat* (3M, Maplewood, MN, USA) on a small Printed Circuit Board (PCB) with two comb electrodes. A flexible layer printed with *Filaflex* (Recreus Industries S.L, Alicante, Spain) is placed atop this structure. Figure 3 shows a picture of the custom FSR and Figure 4 shows the resulting handle with sixteen integrated force sensors.

### 2.2. Analyzed Variables

**Figure 5:**
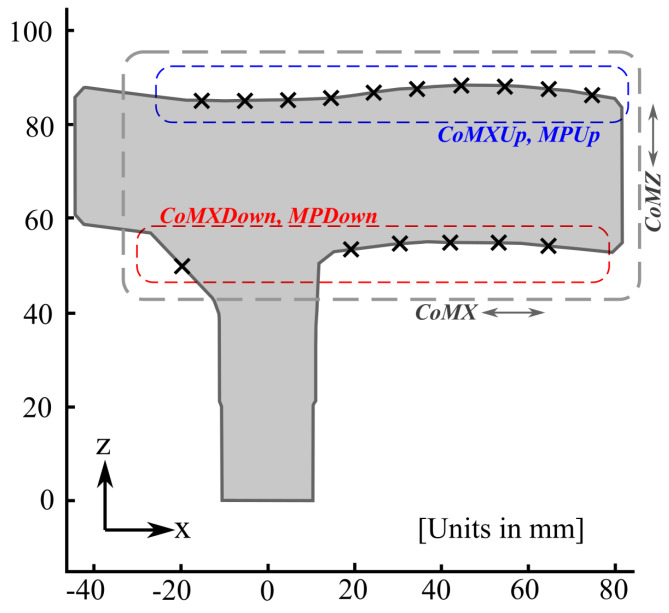
Sagittal view of the handle with the locations of the FSR units marked as crosses. Those under the blue dashed square are used to compute CoMXUp and MPUp; CoMXDown and MPDown are calculated with the ones under the red dashed square. CoMX and CoMZ use the whole set in *x*-axis and *z*-axis directions, respectively.

Six variables plus their time derivatives where examined in this work as possible sources of gait events. Figure 5 shows an illustration of the cane handle with the locations of the sensors with respect to a local reference system in the sagittal plane (*z*-axis is aligned with the shaft of the cane). The coordinates of the locations of the FSRs in this local system are given in Table 1. The evolution of the parameters proposed in this section and their time derivatives are available as Supplementary Files for the tests carried out by all the participants.

Provided that f(i) is the output of the *i-th* sensor, and x(i) and z(i) its coordinates in the local reference of Figure 5, the proposed variables are defined as follows:

- Top side center of mass in *x*-axis direction:
  (1) $$\begin{array}{c} CoMXUp\;=\;\frac{\sum_{i=1}^{N_{Up}}f(i)x(i)}{\sum_{i=1}^{N_{Up}}f\left(i\right)} \end{array}$$
  where the index *i* is the same as that in Table 1 and $N_{Up}\;=\;10$ (number of FSRs on the handle top side).
- Bottom side center of mass in *x*-axis direction:
  (2) $$\begin{array}{c} CoMXDown\;=\;\frac{\sum_{i=N_{Up}+1}^{N_{Up}+N_{Down}}f(i)x(i)}{\sum_{i=N_{Up}+1}^{N_{Up}+N_{Down}}f\left(i\right)}f\left(i\right) \end{array}$$
  where $N_{Down}\;=\;6$ (number of FSR units in the handle bottom side).
- Center of mass in *x*-axis direction:
  (3) $$\begin{array}{c} CoMX\;=\;\frac{\sum_{i=1}^{N_{Up}+N_{Down}}f(i)x(i)}{\sum_{i=1}^{N_{Up}+N_{Down}}f\left(i\right)} \end{array}$$
- Center of mass in *z*-axis direction:
  (4) $$\begin{array}{c} CoMZ\;=\;\frac{\sum_{i=1}^{N_{Up}+N_{Down}}f(i)z(i)}{\sum_{i=1}^{N_{Up}+N_{Down}}f\left(i\right)} \end{array}$$
- Top side mean pressure:
  (5) $$\begin{array}{c} MPUp\;=\;\frac{\sum_{i=1}^{N_{Up}}f(i)}{N_{Up}} \end{array}$$
- Bottom side mean pressure:
  (6) $$\begin{array}{c} MPDown\;=\;\frac{\sum_{i=N_{Up}+1}^{N_{Up}+N_{Down}}f(i)}{N_{Down}} \end{array}$$

## 3. Materials

The different elements involved in the experiment are explained below. They are the instrumented cane, the gait segmentation reference system and the experimental setup.

### 3.1. Instrumented Cane

**Figure 6:**
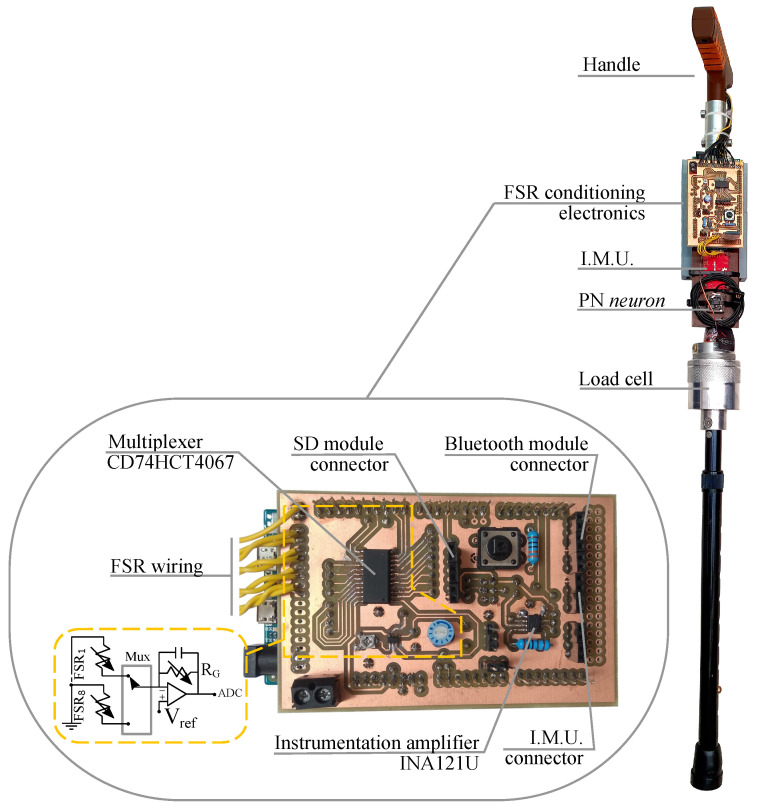
Instrumented cane and detail of the custom shield.

Figure 6 shows the instrumented cane that has been developed to obtain the results of this paper. The handle equipped with sixteen FSRs has been described in Section 2.1. Two more sensors are added to the cane. A load cell that is embedded in the shaft and measures the force along its axis, and a 9DOF IMU (SparkFun Electronics, Boulder, CO, USA) that incorporates a gyroscope, an accelerometer and a magnetometer. The electronics is based on an Arduino DUE. A custom Arduino compatible shield was built to host the analog part of the signal conditioning of the sensors (see Figure 6 detail).

The circuit for the FSRs is based on a transimpedance amplifier that converts changes in the sensor resistance into voltage changes and provides the appropriate gain before the analog-to-digital conversion. A multiplexer (CD74HCT4067 by Texas Instruments, Dallas, TX, USA) is used to select a specific FSR of the array. Moreover, the load cell output is conditioned by a circuit based on an instrumentation amplifier (INA121U by Texas Instruments, Dallas, TX, USA) while the IMU has a digital output provided by the on board ATMega328 microcontroller (Microchip Technology Inc., Chandler, AZ, USA) and is connected to the Arduino via a serial interface. The sensors are scanned at a rate of 50 Hz and its information is digitized in the Arduino and sent to a computer via Bluetooth through the module HC-05 (SparkFun Electronics, Boulder, CO, USA) at 115,200 Bd. The whole system is powered by a battery attached to the shaft. The height of the cane can be regulated.

### 3.2. The Reference System

The motion capture system Perception Neuron (PN) (Noitom Limited, Miami, FL, USA) has been used as reference for the segmentation of the gait. The specific model used for the experiments in this paper is *PN2.0*. This system is based on the so-called *neurons* that are basically inertial units with accelerometers, gyroscopes and magnetometers. Seventeen *neurons* are attached to the segments of the body while one neuron is located on the shaft of the cane (see Section 3.3 for the location of the *neurons*). Each neuron provides position, speed, quaternions, acceleration and angular velocity. The technical specifications of these sensing units are gathered in Table 2.

**Figure 7:**
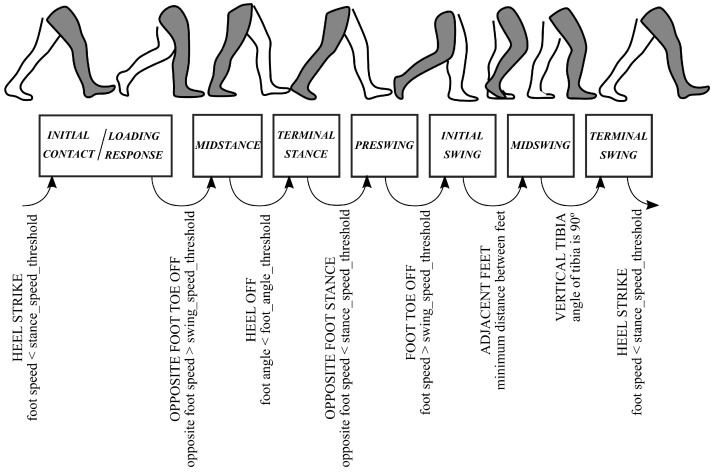
Events used for reference gait segmentation with the information provided by the Perception Neuron.

Figure 7 shows the algorithm that has been followed to split the stride into eight gait phases. For an eight phase granularity, the *initial contact* phase takes about the 8% of the gait cycle, so it has been merged with the *loading response* phase in this study. The parameters that have been used in this work are: stance_speed_threshold = 0.4 m/s, swing_speed_threshold = 0.8 m/s and foot_angle_threshold = −9°. The condition of adjacent feet is signaled when the distance between both feet is a minimum and is lower than a distance threshold of 0.4 m (this rule is added to increase the robustness of the detection). Finally, the condition of vertical tibia is given when the corresponding *neuron* provides an orientation of 90° with respect to the horizontal axis.

### 3.3. Experimental Setup

**Figure 8:**
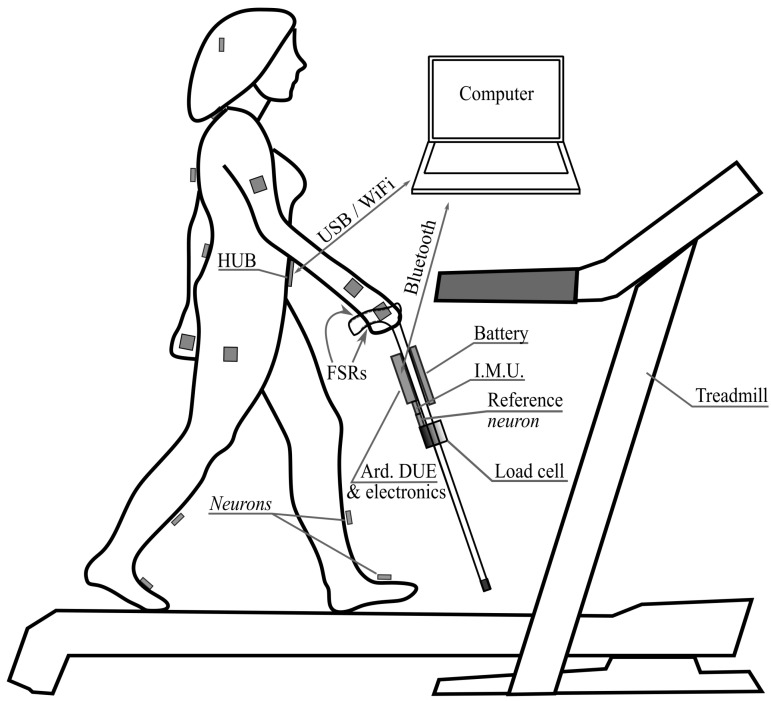
Experimental setup.

Figure 8 depicts the experimental setup that has been used to gather the data and obtain the results that will be presented in Section 5. The volunteer is equipped with the inertial sensing units (*neurons*) of the reference motion tracking system Perception Neuron. Another *neuron* is attached to the shaft of the cane, close to its own IMU, for synchronization purposes (see Section 4.3). The data collected by the PN units are sent to a hub in the belt and then to a computer via USB or WiFi. The instrumented cane described above sends also the information collected by its sensors to the computer, via Bluetooth in this case. A treadmill that allows the volunteer to walk at constant speed while using the cane completes the setup.

## 4. Methods

This section covers the experiment methodology. It focuses on the group of participants, the experiment protocol and the captured data processing.

### 4.1. Participants

The experiment involved 12 healthy individuals [P1–P12]. They were 9 males and 3 females, aged between 25 and 50 years old, 33.1 on average and a SD of 9.2 years. The study was carried out in conformity with the Declaration of Helsinki of the World Medical Association, and all the participants gave their informed consent. Their gait was altered by wearing a modified insole in one of their shoes to simulate a slight walking impairment and encourage the use of the cane.

### 4.2. Protocol

The volunteers were asked to wear comfortable clothing and sport shoes. They held the instrumented cane in a contralateral way, after adjusting its height to their body characteristics. While walking, the cane motion was synchronized with the impaired leg, so that it provided proper support. In the experiment, the participants walked on the treadmill at 1.8 m/s. During the experiment design phase, speeds of 1.4 m/s, 2.2 m/s and 2.6 m/s were also tested. The chosen speed was that found most comfortable by volunteers.

**Figure 9:**
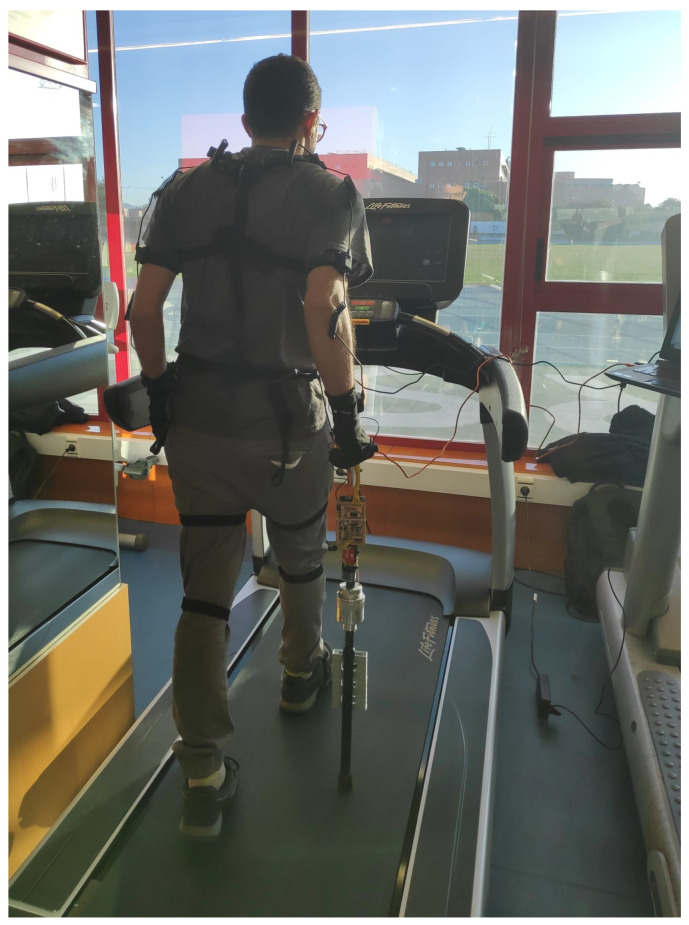
Volunteer taking part in the preliminary trials during the experiment design.

Before capturing data, they walked for some minutes on the treadmill to become familiar with the cane. Then, they were equipped with the 17 neurons of the PN system to register the gait. With the setup ready, they walked on the treadmill for two minutes. Figure 9 shows a volunteer during the trials of the experiment design.

### 4.3. Data Processing

Once the data capturing is done, the first step in the data processing is the synchronization of the signals. Moreover, the *neurons* involved in the capture of the lower limbs pose, an extra *neuron* of the Perception Neuron system is attached to the shaft of the cane. Its purpose is performing the synchronization between the Perception Neuron and the instrumented cane by aligning the received data from the cane IMU and the cane PN *neuron*, since they should provide the same information (a similar procedure is followed in [28] to synchronize a Vicon motion system (Vicon Motion Systems Ltd., Oxford, UK) with an inertial sensors-based system). To carry out this task the angles $\alpha_{cane}$ and $\alpha_{PN}$ between the cane and the vertical axis with respect the floor have been obtained from the data given by both components. Then, the following procedure is performed:

1. $\alpha_{cane}$ and $\alpha_{PN}$ are normalized to obtain their output between 0 and 1.
2. The portion of both signals that precedes or is after the first and last local peak respectively, is removed.
3. Since the scanning rate of the PN is higher than that of the instrumented cane, more samples are obtained by interpolation for the latter to obtain the same number for both.
4. One of the resulting signals is shifted with respect the other until a maximum Pearson correlation between the two of them is obtained.
5. The shift between both signals computed in the previous step is stored and added to all signals from the cane and the PN to synchronize them.

Once the signals are synchronized, high frequency and noise peaks are removed with a 3rd order Savitzky-Golay filter. With the purpose of increasing the regularity in the signals, the 30 central strides of each test are used to study the possible gait events. The variables of Section 2.2 are computed for each gait cycle, and maximums and minimums are detected. A subsequent exploration of the evolution of the parameters is carried out to find if there are events that can be useful either to locate the beginning of a gait phase or to anticipate its arrival.

## 5. Results and Discussion

**Figure 10:**
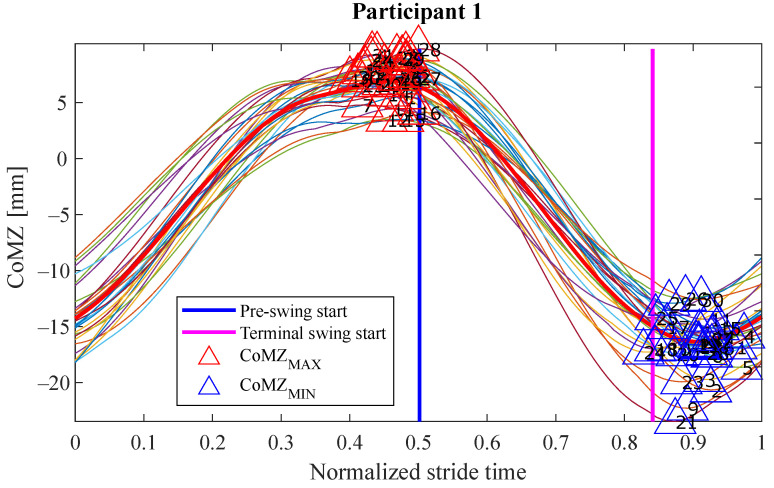
Evolution of the variable CoMZ for the test carried out by P1. The duration of the strides has been normalized to ease the visual inspection (*initial contact* takes place at 0 in *x*-axis and the stride finishes at 1). The red line is the mean of CoMZ considering all the gait cycles involved. The vertical lines represent the mean time in which those phases started. In this case, gait events can be seen just before the *pre-swing* phase and during the *terminal swing* phase, in form of maximums and minimums of CoMZ, respectively. The former may be used to anticipate the *pre-swing* phase and the latter to do the same with the *initial contact* of the following stride (it would take place at 1 in *x*-axis).

The experiments results are shown below in box plots for all the participants involved. The box plots gather the times in which relevant maximums or minimums of the studied variables appeared, taking as reference the beginning of the different gait phases provided by the PN system for each stride. This way, 0 s represents the phase start, negative values of time mean that the event occurs before the phase and positive values mean that it appears once the phase has started. Only the events that appeared at least in 25 out of the 30 strides were included. The prefix *d* is used to indicate that the parameter is the derivative with respect to time of one of the variables explained in Section 2.2 (for example, dCoMZ is the derivative of CoMZ). Figure 10 helps visualize how variables change over time during the gait cycles and how potential events appear.

**Figure 11:**
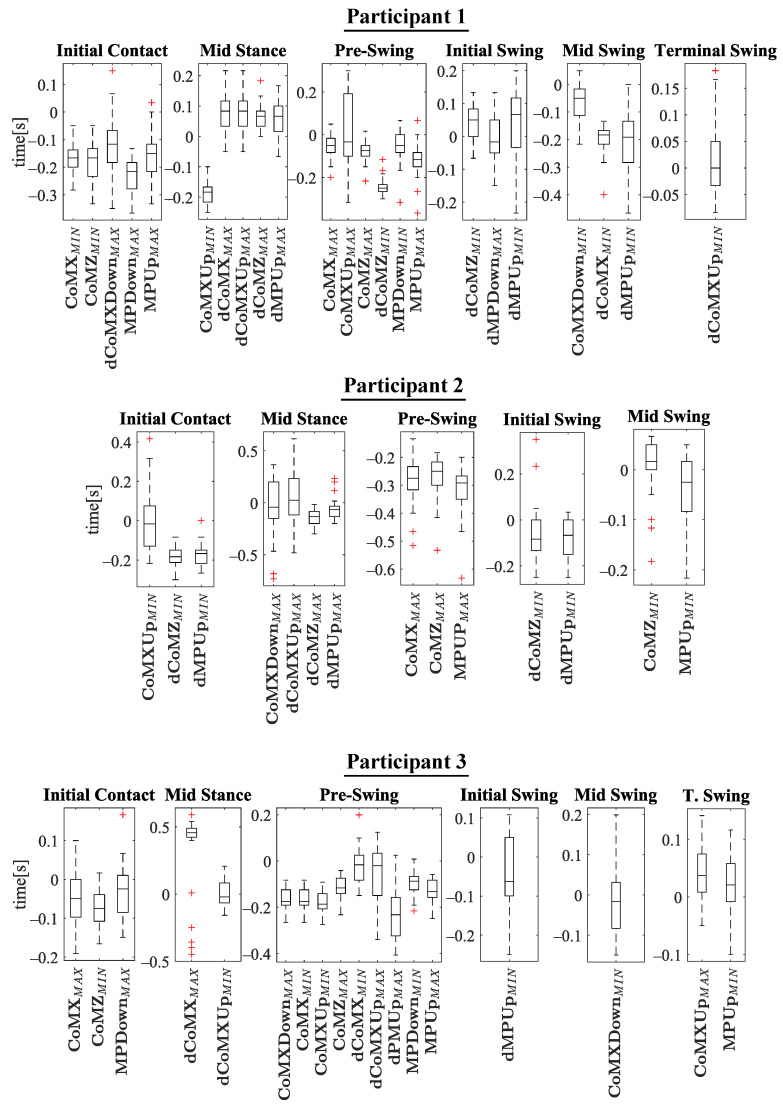
Experimental results for P1–P3. In the *y*-axis, 0 s is the time corresponding to the phase beginning. Negative time values mean that the event appears before the phase, and positive values that the it occurs after the phase has started.

**Figure 12:**
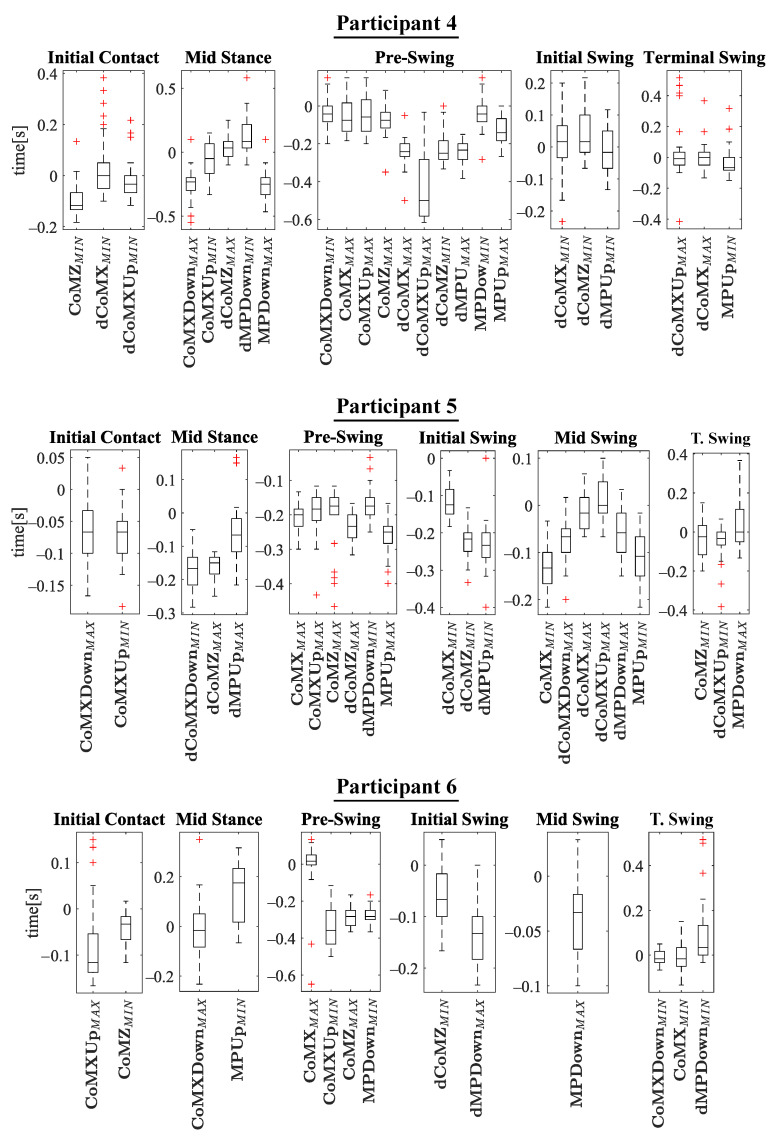
Experimental results for P4–P6. In the *y*-axis, 0 s is the time corresponding to the phase beginning. Negative time values mean that the event appears before the phase, and positive values that the it occurs after the phase has started.

**Figure 13:**
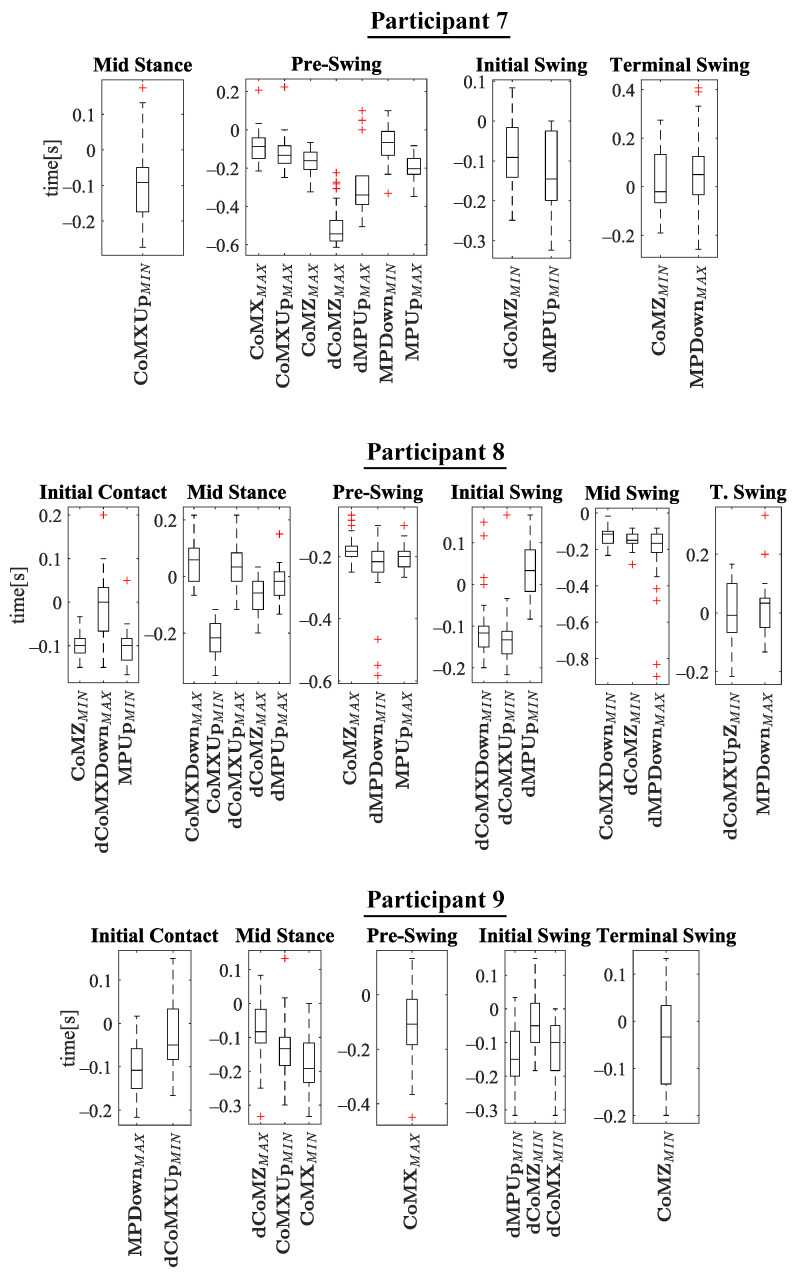
Experimental results for P7–P9. In the *y*-axis, 0 s is the time corresponding to the phase beginning. Negative time values mean that the event appears before the phase, and positive values that it occurs after the phase has started.

**Figure 14:**
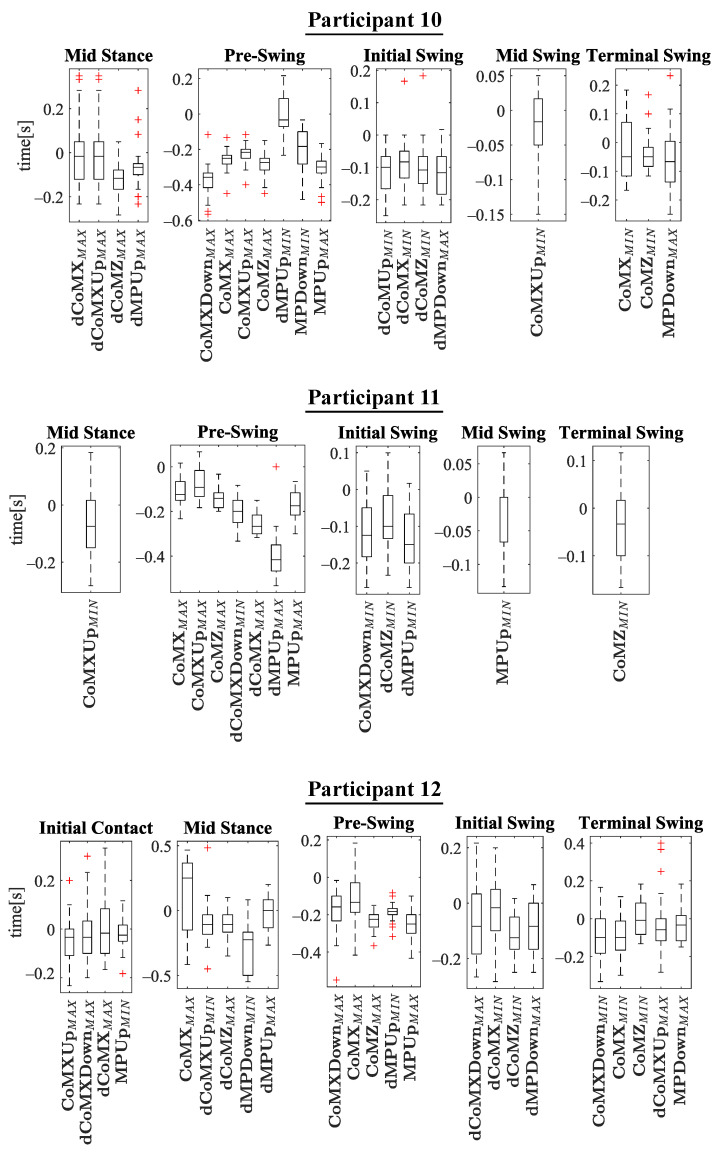
Experimental results for P10–P12. In the *y*-axis, 0 s is the time corresponding to the phase beginning. Negative time values mean that the event appears before the phase, and positive values that the event occurs after the phase has started.

**Figure 15:**
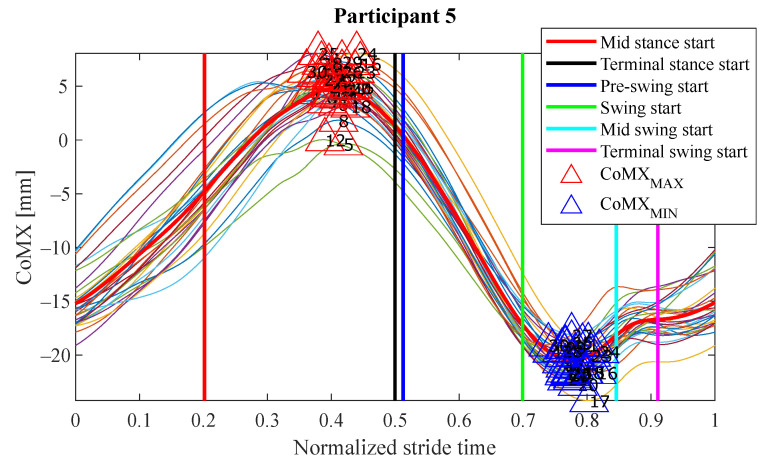
Evolution of the variable CoMX for the test carried out by P5. As can be seen, the event formed by the CoMX maximums may be used both to anticipate *terminal stance* or *pre-swing* phases, due to the proximity between the two of them.

Please note that the phase *terminal stance* is not included in Figure 11, Figure 12, Figure 13 and Figure 14. On the one hand, there was certain lack of accuracy in our gait segmentation system for this phase detection, that did not allow identifying it properly for a few participants. On the other, although it was well identified for the rest, it was so close to *pre-swing* phase that there were no enough time to have gait events between the two of them. For this reason, it was decided not to include the *terminal stance* phase in the results and to use only *pre-swing* as reference. Figure 15 shows that the gait events found before *pre-swing* may be used as well to anticipate *terminal stance* due to the short time interval between the two phases. Apart from explained above, when other phases do not appear in Figure 11, Figure 12, Figure 13 and Figure 14 is just because no relevant gait events were found for them in the test of that participant.

The main goal of this research is looking for events provided by FSRs mounted on the handle of a cane that signalize the gait and could be exploited to improve the control of prostheses or FES. Although the reliability of FSRs is low when estimating the magnitude of the force, they perform well to provide temporal information that is involved in real-time event detection [3]. Results in Figure 11, Figure 12, Figure 13 and Figure 14 show many detected peaks to be used as gait events of the examined variables defined in Section 2.2. All of them present either a maximum or minimum that signalize or anticipate the arrival of a phase.

**Figure 16:**
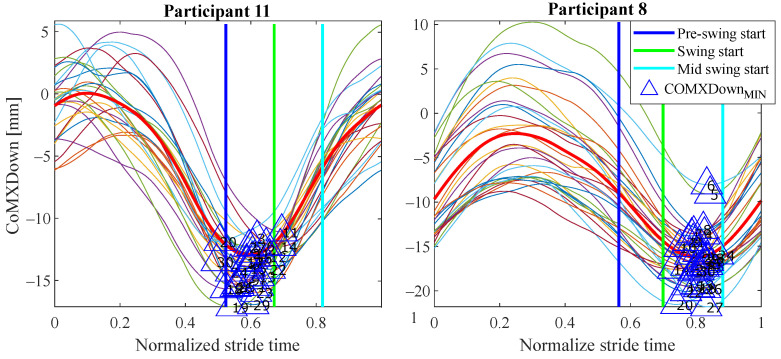
Evolution of the variable CoMXDown for the tests carried out by P8 and P11. A time shift can be observed in the variable when comparing the tests. As a result, the event formed by $CoMXDown_{MIN}$ anticipate a different phase for one volunteer and the other.

As can be seen, some events appear in the sames phases for many of the 12 participants, for example: $CoMX_{MAX}$ for 10 volunteers, $CoMZ_{MAX}$ for 11 and $MPUp_{MAX}$ for 10 in *pre-swing*; $dCoMZ_{MAX}$ for 8 participants in *mid stance*; $CoMZ_{MIN}$ for 10 and $dMPUp_{MIN}$ for 10 in *initial swing*; or $CoMZ_{MIN}$ for 7 in *terminal swing*. Despite this, a significant inter-subject variability is observed. Several reasons may be behind it. For example, some of the variables are slightly time shifted between participants. This makes the same variable peak appear as gait event in different consecutive phases when comparing the tests. This is illustrated in Figure 16, where CoMXDown is shifted in time axis for P8 with respect to P11. This way, even if the evolution of the parameter is similar, in one case the event $CoMXDown_{MIN}$ anticipates the *swing* phase and in the other *mid swing*. This may be explained by slight differences in the *impaired leg − cane* synchronization of the two volunteers (e.g., for one of the users, there may be a small angular lag between the leg and the cane when moving).

**Figure 17:**
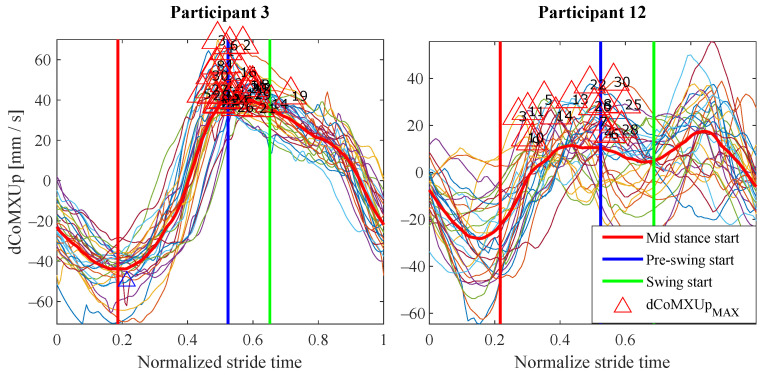
Evolution of the variable dCoMXUp for the tests carried out by P3 and P12. For the latter, the maximums of the variable surrounding *pre-swing* are distributed in a time interval so large that an accurate phase detection would not be feasible.

This variability implies that any gait detection procedure that exploited this information should be adapted to the user. Inter-subject variability is common in people with different walking handicaps and it is also influenced by the assistive device [2,3,29]. Moreover, although a previous explanation was given to the volunteers and some practice was allowed, they had no experience in the use of canes and some of them could likely do it in a wrong way. This could explain some poor results observed for example for P12 in Figure 17, when compared with those from other volunteers such as P3. As can be observed, the trajectories of dCoMXUp are quite erratic for the strides of P12 test. This way, whereas there are maximums that may allow detecting the *pre-swing* start in P3 test, in the curves of dCoMXUp for P12, the maximums surrounding *pre-swing* cover a wide segment of time, what disables them as useful gait events for detection. This performance can be noted as well in Figure 14, for example, when comparing the time dispersion of event $CoMX_{MAX}$ in *mid stance* for P12, with that obtained for the events of other subjects in the same phase. A larger time dispersion implies less reliable and accurate gait events.

Actually, the improper use of the cane seems to be common in many of its users, as reported in [8]. Thus, this point should be taken into account in the case of implementing an assistive system based on the readings of sensors in the instrumented cane. Please note that a high number of events can be signalized with the proposed device. This can help detect a number of sub-phases, which can be beneficial for lower extremity orthoses, prostheses and FES walking systems [30]. Heel strike and toe off (*initial contact* and *initial swing* in Figure 7, respectively) are the most sought-after gait events [3]. For example, the Myosuit [31] could use the early detection at heel-strike to pre-tension the system for the upcoming force application. In direct comparison, the late toe-off detection can cause problems [16]. In addition, detection of all *swing* phases is critical in applications such as FES [30] (as can be observed in Figure 11, Figure 12, Figure 13 and Figure 14, all of them are detected for *swing* for P1, P3, P5, P6, P8, P10 y P11). Moreover, intermediate points between the standard gait events may be important in control of assistive systems [26] (for example, $CoMX_{MAX}$ in Figure 15).

Many events in the results anticipate a given phase (time values below zero in Figure 11, Figure 12, Figure 13 and Figure 14). These events can be found in the tests of all the subjects for which *initial contact* is detected; in the tests of P1, P2, P4, P5, P7, P8, P9, P10 and P12 for *mid stance*; for all the volunteers in *pre-swing* phase; in the tests of P2, P5, P6, P7, P8, P9, P10, P11 and P12 for *initial swing*; in those of P1, P5, P6, P8 and P11 for *mid swing*; in the tests of P4, P5, P10 and P12 for *terminal swing*. The anticipation is interesting because it is necessary to compensate the delay of electromechanical devices in prostheses (30–100 ms reported in [32]), or the time it takes the muscles to develop sufficient force after electrical stimulation in FES (50–150 ms in [33,34]). However, the temporal location of a peak along the gait cycle can be better seen as a means to characterize it and signalize the main events, but it does not limit the possibility of obtaining usable beacons that precede the gait phases even if the peak itself does not anticipate the phase. Actually, different authors have reported algorithms that are able to anticipate the main phases with inertial sensors [2,16]. Specifically, rules-based and machine learning detection algorithms can exploit the repeatability of the gait cycle for this purpose. Rule-based algorithms are more appropriate for real time operation and also are more flexible to be implemented in cases of unilateral assistance, for instance [3].

Regarding the event detection, most real time algorithms are threshold-based, i.e. the event is detected when the tracked signal crosses a certain threshold [29]. But the obtained delays with respect to the reference system depends on the choice of the threshold itself [2,35], and even the reference borders can be affected for the same reason. The comparison between a system based on FSR and the Vicon indicates that the difference data is widely distributed in experiments with child with Cerebral Palsy in [26]. In this sense, events with low intra-subject variability, such as those specified above for Figure 11, Figure 12, Figure 13 and Figure 14, are valuable. For instance, a time no earlier as well as no later than 100 ms the reference system signals the heel off is required for correct control of a drop foot stimulator [35]. A potential real time application is related to the determination of the location of the segments of a lower limb exoskeleton while walking. Some works have been proposed in this regard [36]. The proposal of this paper may be useful in this task as a secondary source of information for drift correction.

The results presented in this paper are promising in the sense that they confirm the proposed strategy as feasible to obtain valuable events along the gait cycle. However, there are some limitations that should be faced in future works. First, although an insole is used to simulate certain dysfunction and force the use of the cane, the experiments have been performed with healthy subjects. This is quite common in reported works about gait [29], but it certainly means that further experiments with handicapped people should be carried out. The obtained results can be more easily applied to people that retain the ability to walk although being handicapped, and need the help of a soft exoskeleton, for instance [18,19]. However, the large variability of health problems and subject gait characteristics points to custom subject oriented gait detection algorithms. The detection of gait phases is also strongly dependent on the environmental conditions such as different terrains as well as the walking speed. In summary, the events and the thresholds can depend on the subject and the conditions [35,37]. Still these are real life conditions so effective gait detection systems should take them into account. In this sense, the addition of more sensors to perform sensor fusion is a way to have success in this task [4]. A combination of inertial sensors with footswitches and foot pressure insoles is mentioned in [29] as a way to improve the robustness of the gait detection algorithm and increase the granularity or resolution, so that more sub-phases are recognized.

As stated in the introduction, this work focuses on a system where the subject has only one affected side and uses a cane. Moreover, the goal is that none sensor is located on the body of the subject, only on the exoskeleton and the cane. Taking into account that complex machine learning algorithms to increase the granularity are not suitable for real time control, the use of more sources of information such as the device of this paper is a way to improve the performance of the control of assistive devices. In the end, it is necessary to find out the right combination of sensors and gait detection methods [3] and the device of this paper can be a good candidate for this purpose.

## 6. Conclusions

The results of this paper show that force sensing resistors mounted on the handle of an instrumented cane provide useful information about gait that can be exploited in efficient control of prostheses, exoskeletons, or Functional Electrical Stimulation systems. However, a large inter-subject variability is observed, so specific systems with chosen variables and events must be developed for each impaired person. Many events anticipate the onset of subphases. This is observed and especially interesting in the case of the initial contact subphase and the pre-swing subphase, being the beginning of the stance and swing phases the most sought-after gait events. Several users present events in all phases of the swing phase also, which is said by some authors as key in these assistive systems.

A high number of events are obtained for most volunteers. Even if they themselves do not anticipate a given subphase, they signalize intermediate points along the gait cycle and can used to anticipate it, as demonstrated by other authors with inertial sensors. This can be carried out with proper threshold tuning and rules (better for real-time control) or machine learning algorithms. A successful control will likely require the implementation of sensor fusion techniques, for instance with data from the FSRs and the IMU in the cane, but avoiding the location of sensors on the body of the user, to improve the usability and acceptance of the assistive system.

## Figures and Tables

**Table 1:** FSR locations on the handle, according to the local reference of Figure 5.

FSR Unit	X Location [mm]	Z Location [mm]
1	−15.21	85.05
2	−5.29	84.95
3	4.71	85.22
4	14.58	85.61
5	24.44	86.72
6	34.50	87.72
7	44.60	88.19
8	54.71	88.10
9	64.80	87.47
10	74.86	86.27
11	−19.66	49.89
12	19.12	53.30
13	30.40	54.54
14	42.10	54.85
15	53.15	54.74
16	64.65	54.08

**Table 2:** Specifications of the Perception Neuron sensing units.

*Neuron* Specifications	
Size	12.5 × 13.1 × 4.3 mm
Dynamic Range	360 deg
Accelerometer Range	±16 g
Gyroscope Range	±2000 dps
Resolution	0.02 deg
Static Accuracy:	
Roll	<1 deg
Pitch	<1 deg
Yaw angle	<2 deg

## Data Availability

Not applicable.

## Supplementary Materials

The following are available at https://www.mdpi.com/article/10.3390/s21165632/s1, The evolution of the parameters proposed in Section 2.2 and their time derivatives are available as supplementary files for the tests carried out by all the participants.

## Abbreviations

FSR	Force sensor resistor
FES	Functional Electrical Stimulation
IMU or I.M.U.	Inertial Measure Unit
PN	Perception Neuron
CoM	Center of Mass
MP	Mean pressure
