# Supplementary material for: Generation of Gait Events with a FSR Based Cane Handle
Source: Sensors (Basel). 2021 Aug 21;21(16):5632. doi: 10.3390/s21165632 (PMC8402470; doi:10.3390/s21165632)
Supplement: Supplementary file 1 [file sensors-21-05632-s001.zip › Supplementary material.pdf]

Supplementary material

The figures attached gather the evolution of the studied parameters for the tests carried out by all the participants. They are in Matlab format (.fig).

In *x-axis*, the duration of the strides has been normalized between 0 and 1 to ease the visual inspection. This way, phase *initial contact* takes place at 0 and the stride finishes at 1.

Units in *y-axis* are millimeter for the six parameters and millimeter per second for their time derivatives (*d-* prefix has been used for the latter).

Regarding the curves, the red line is the mean considering all the gait cycles involved. The colored vertical lines represent the mean time in which the phases started (see the legend below).

Finally, the red and blue triangles point at the stride maximums and minimums, respectively.

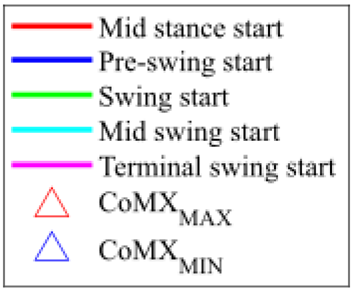

Figure S1. Legend of the curves attached.

The authors
