# Supplementary figures and images for: Generation of Gait Events with a FSR Based Cane Handle
Source: Sensors (Basel). 2021 Aug 21;21(16):5632. doi: 10.3390/s21165632 (PMC8402470; doi:10.3390/s21165632)

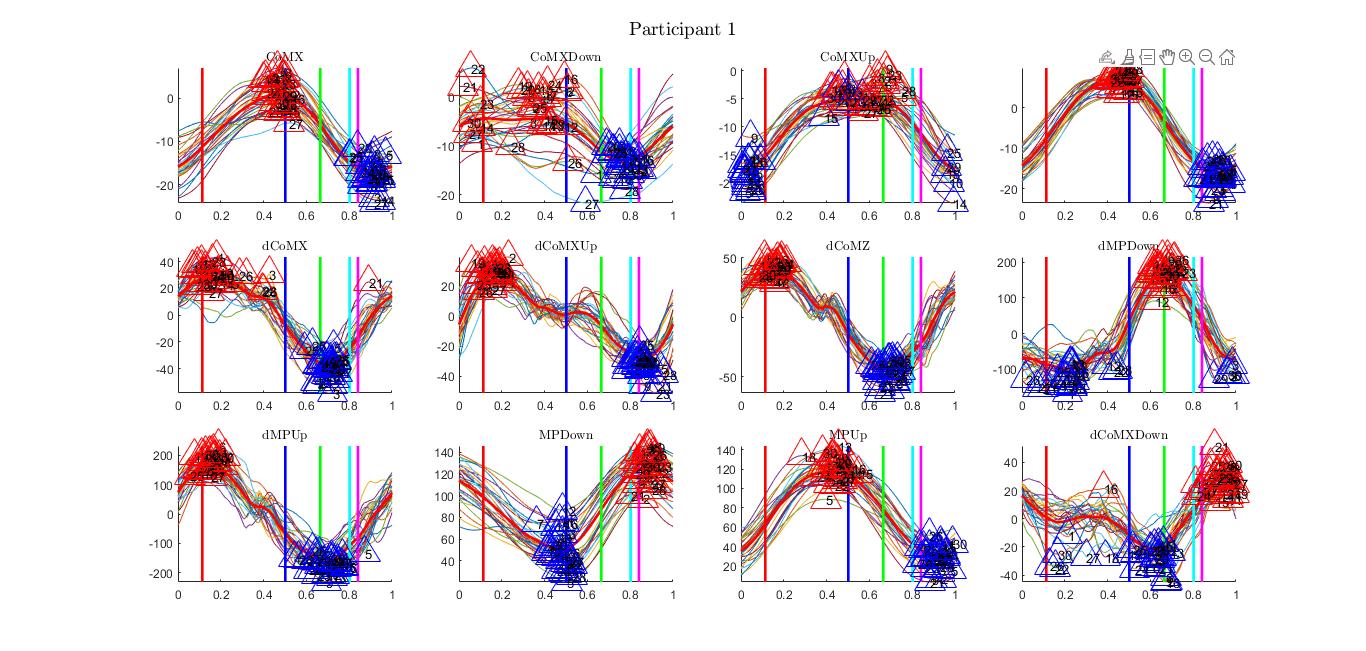

Supplement: Supplementary file 1 [file sensors-21-05632-s001.zip › Participant1.jpg]

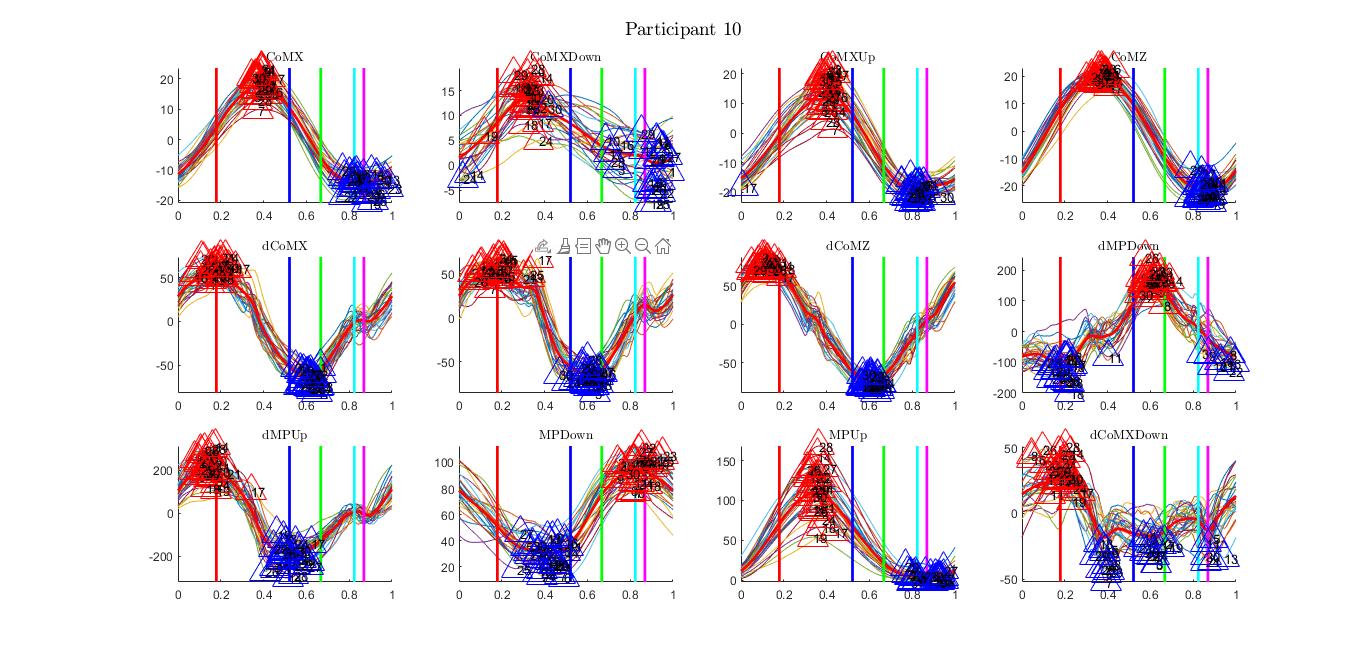

Supplement: Supplementary file 1 [file sensors-21-05632-s001.zip › Participant10.jpg]

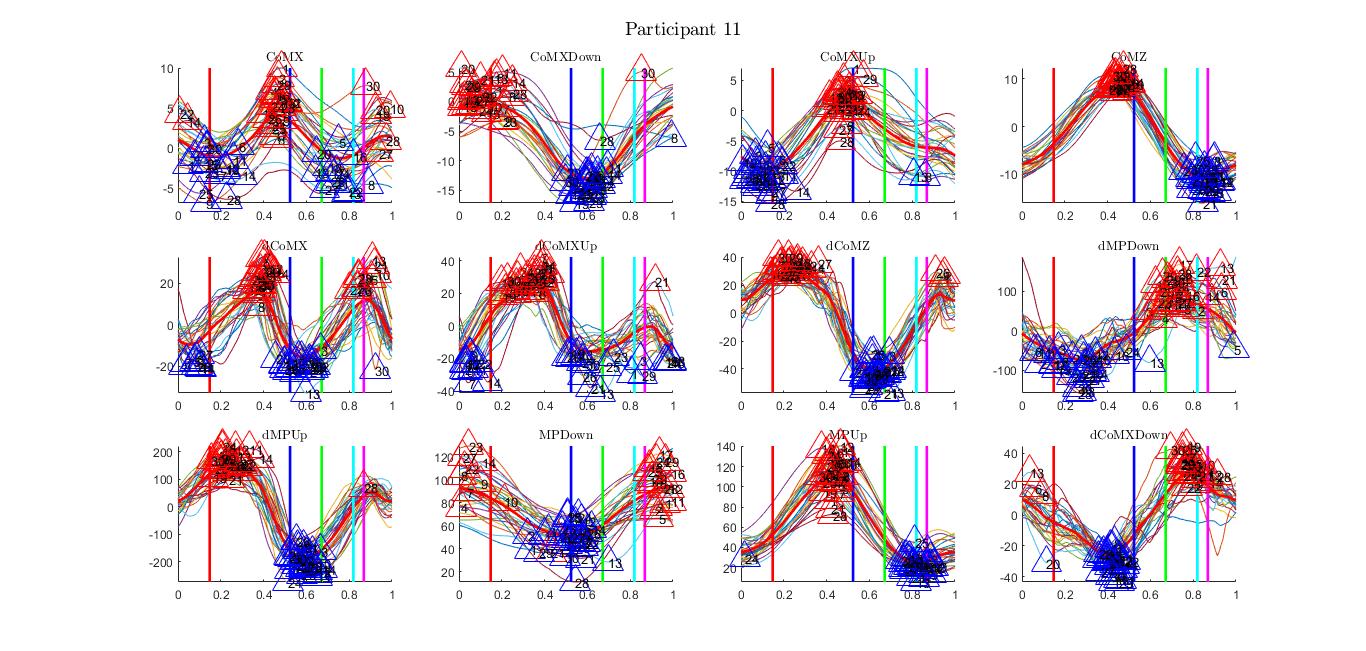

Supplement: Supplementary file 1 [file sensors-21-05632-s001.zip › Participant11.jpg]

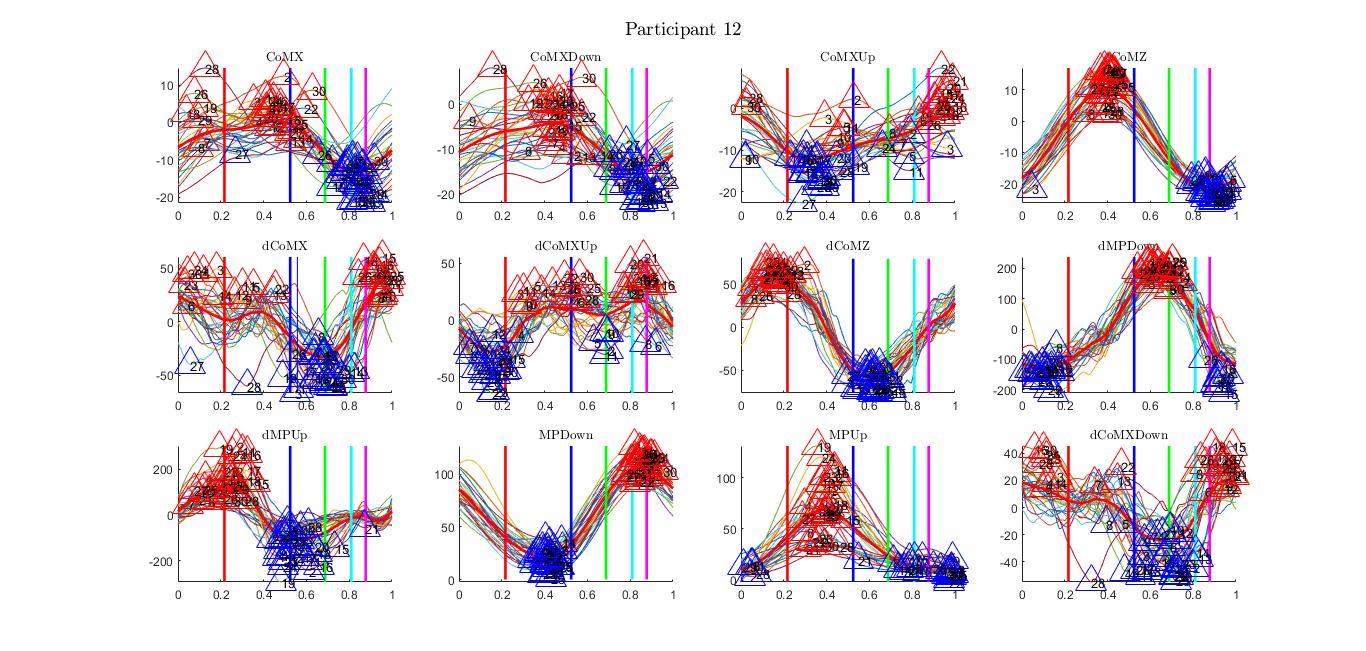

Supplement: Supplementary file 1 [file sensors-21-05632-s001.zip › Participant12.jpg]

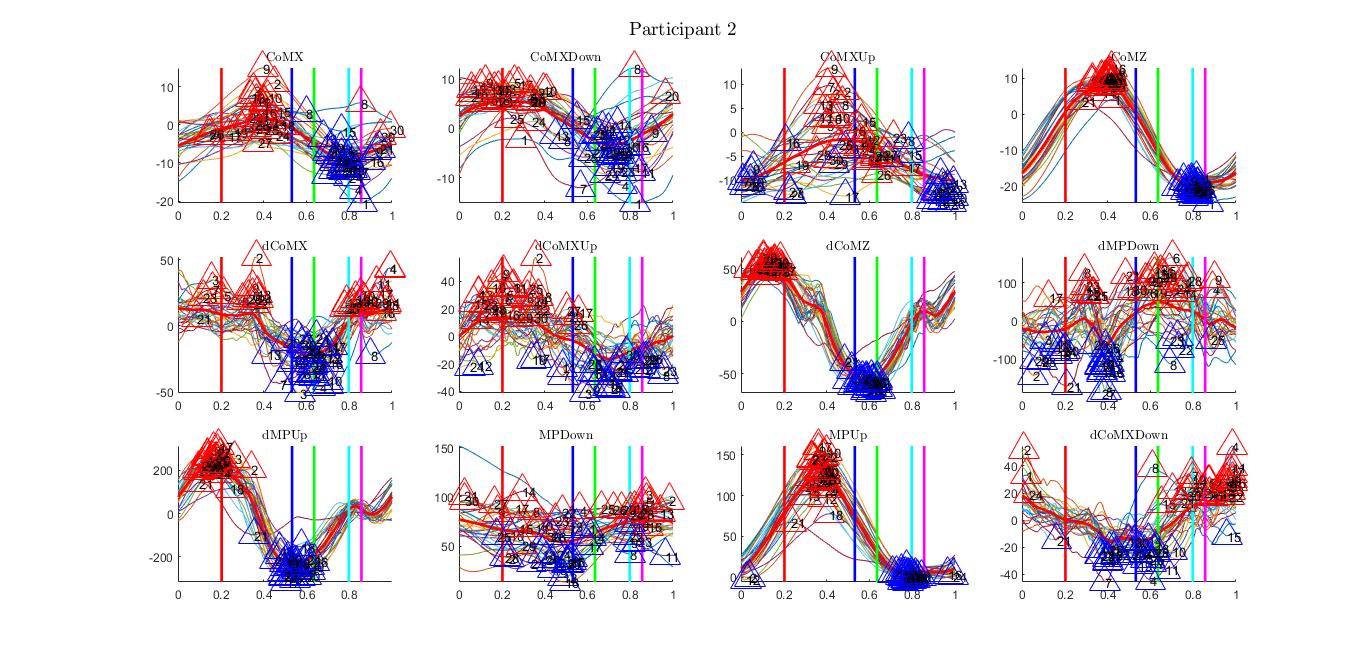

Supplement: Supplementary file 1 [file sensors-21-05632-s001.zip › Participant2.jpg]

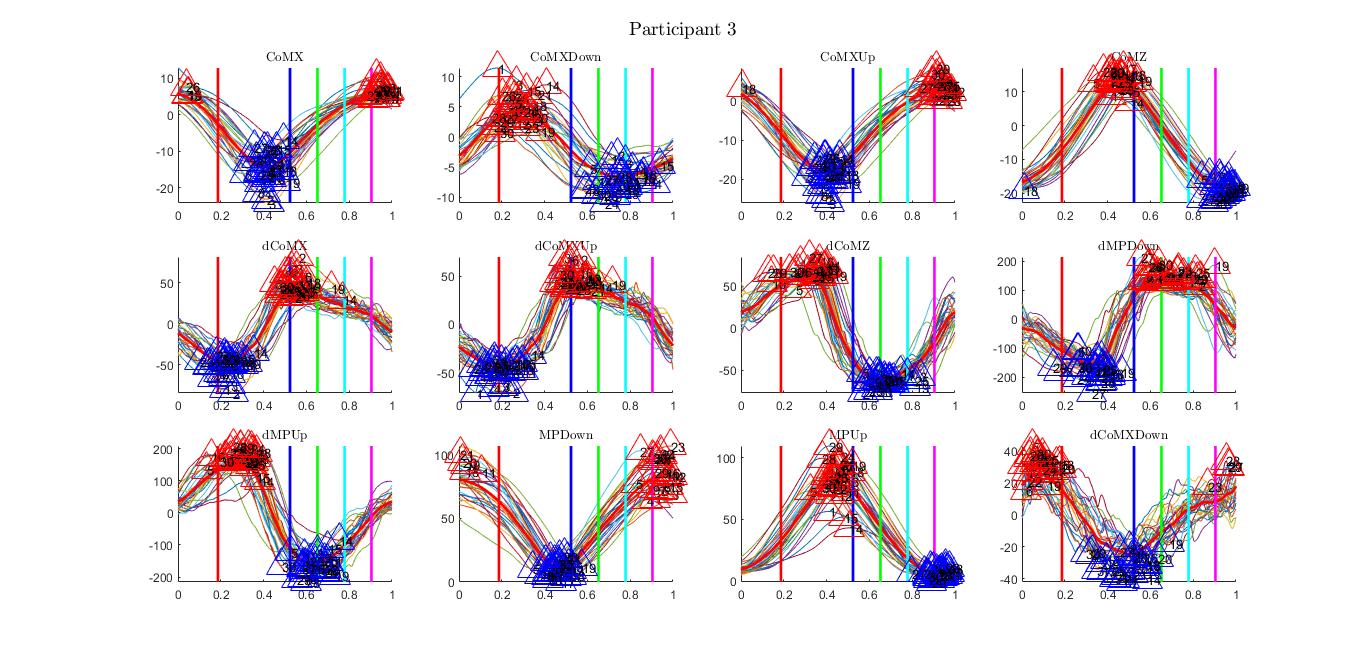

Supplement: Supplementary file 1 [file sensors-21-05632-s001.zip › Participant3.jpg]

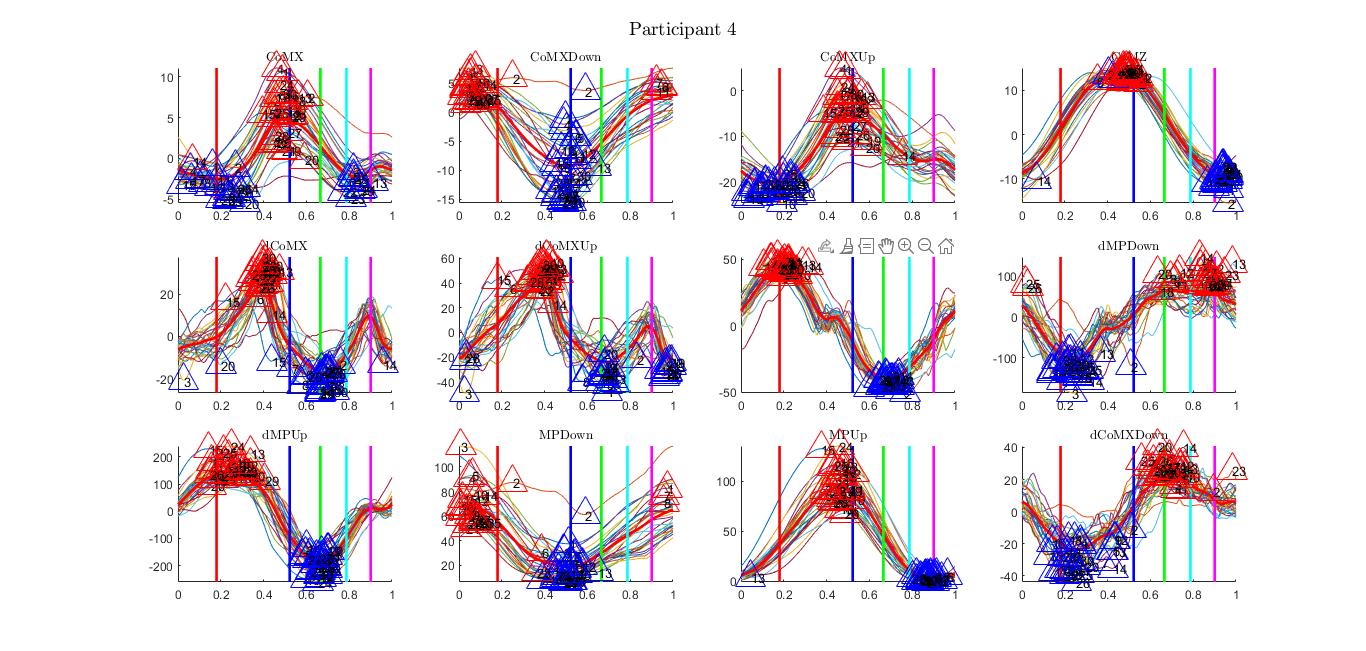

Supplement: Supplementary file 1 [file sensors-21-05632-s001.zip › Participant4.jpg]

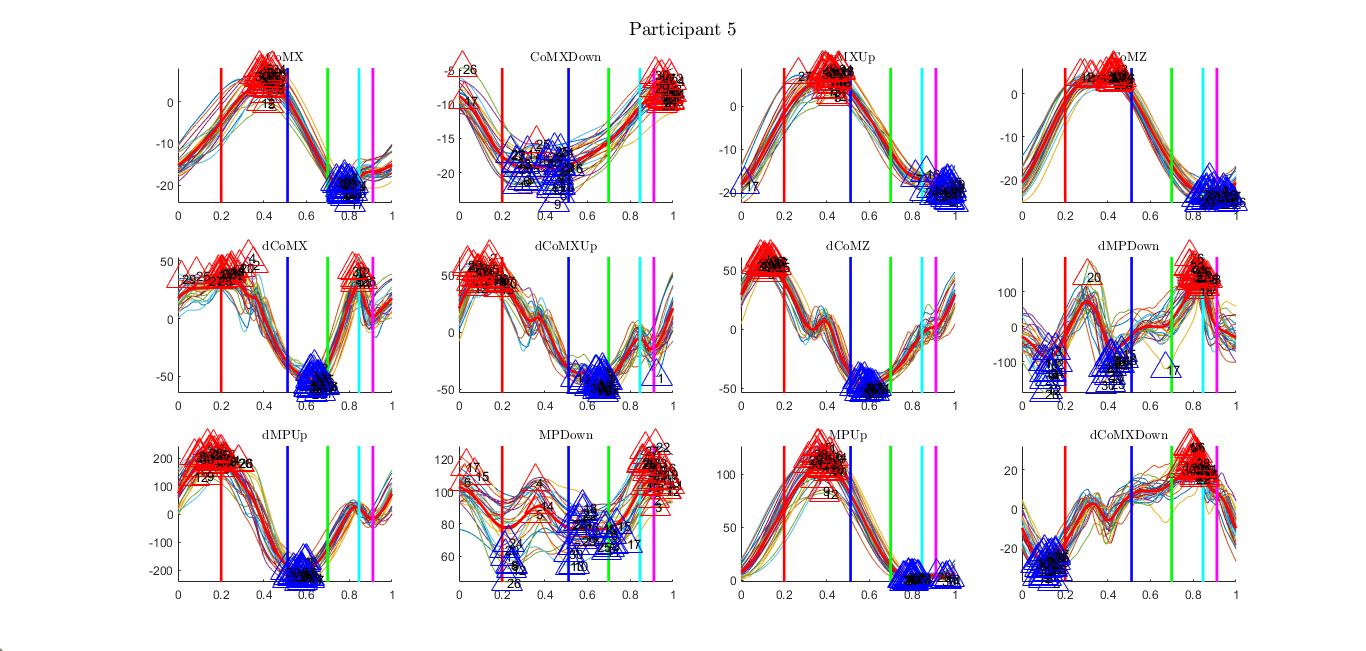

Supplement: Supplementary file 1 [file sensors-21-05632-s001.zip › Participant5.jpg]

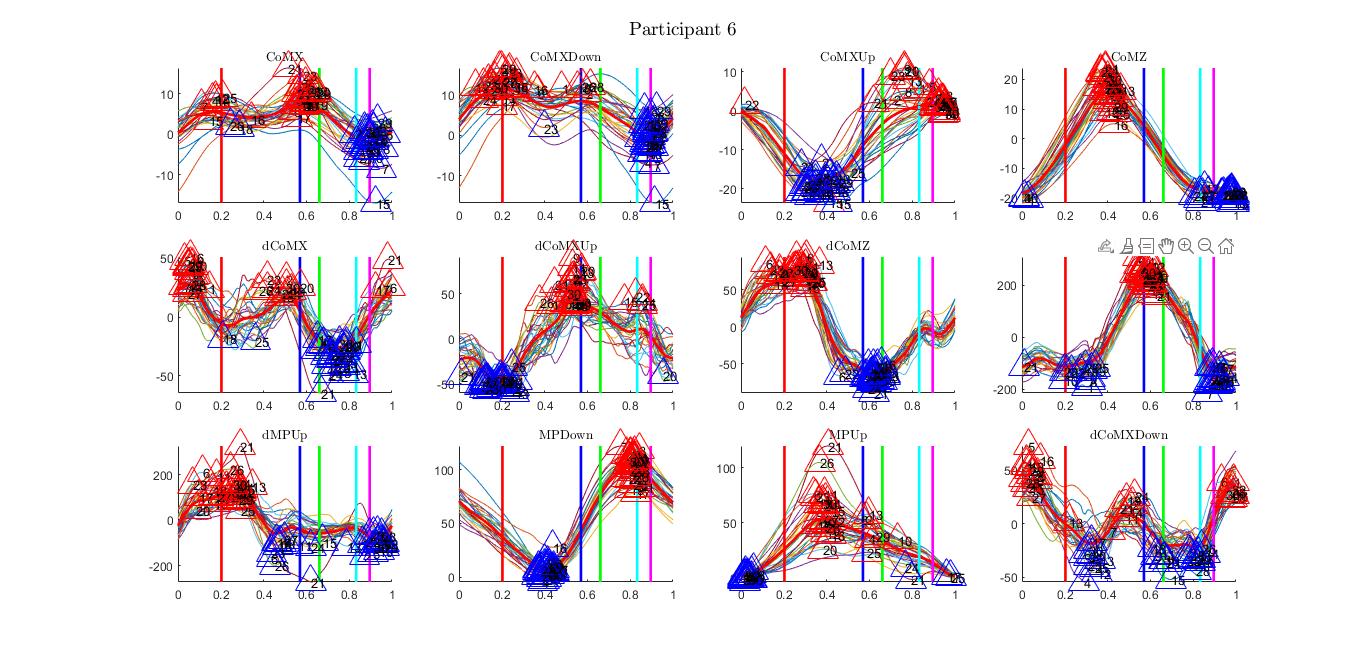

Supplement: Supplementary file 1 [file sensors-21-05632-s001.zip › Participant6.jpg]

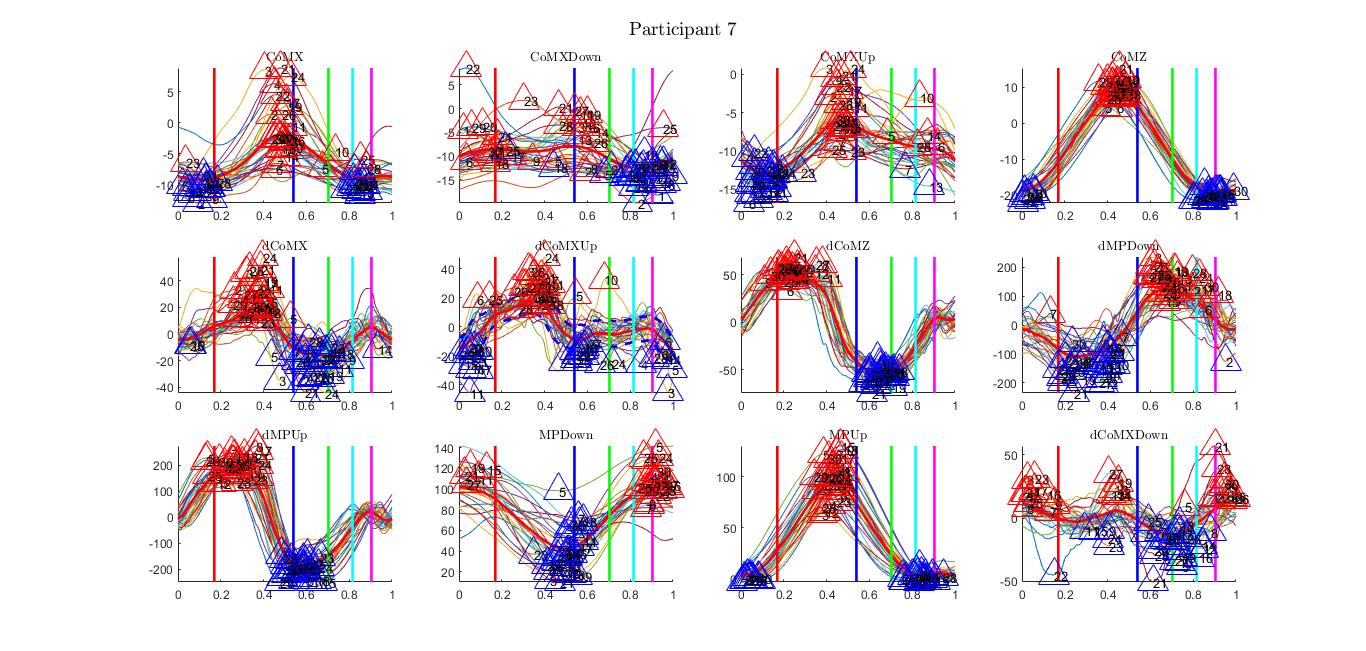

Supplement: Supplementary file 1 [file sensors-21-05632-s001.zip › Participant7.jpg]

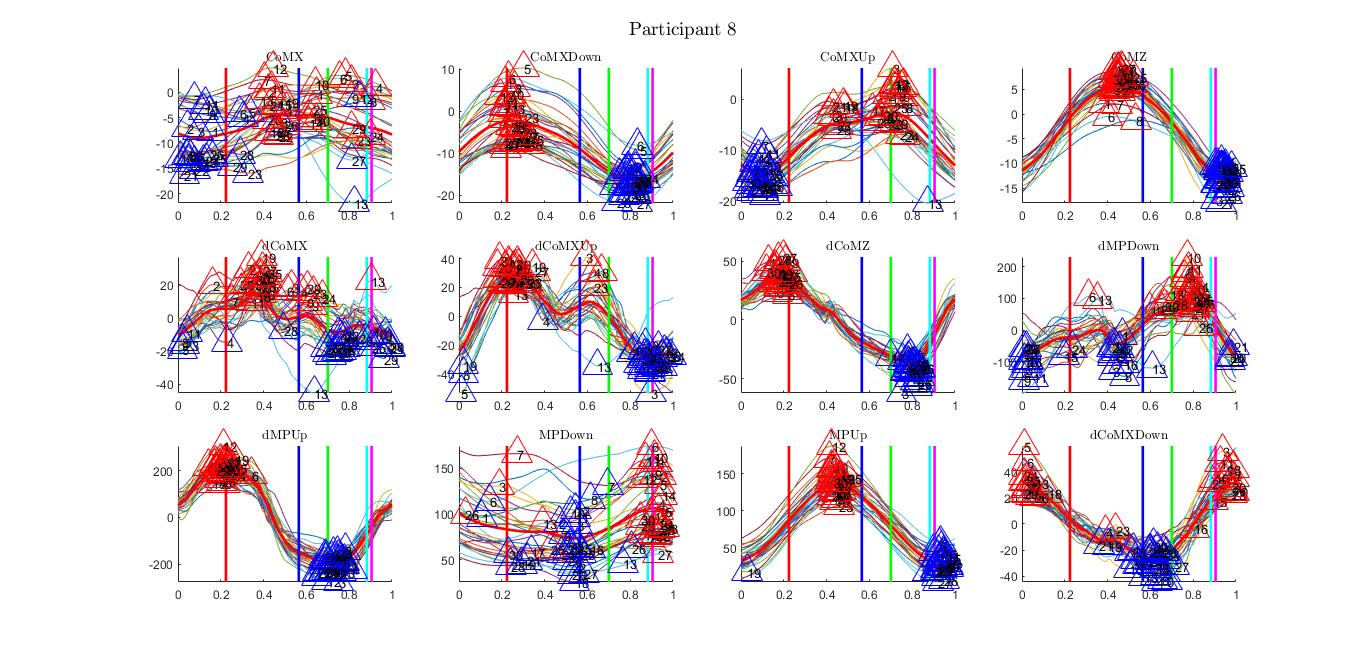

Supplement: Supplementary file 1 [file sensors-21-05632-s001.zip › Participant8.jpg]

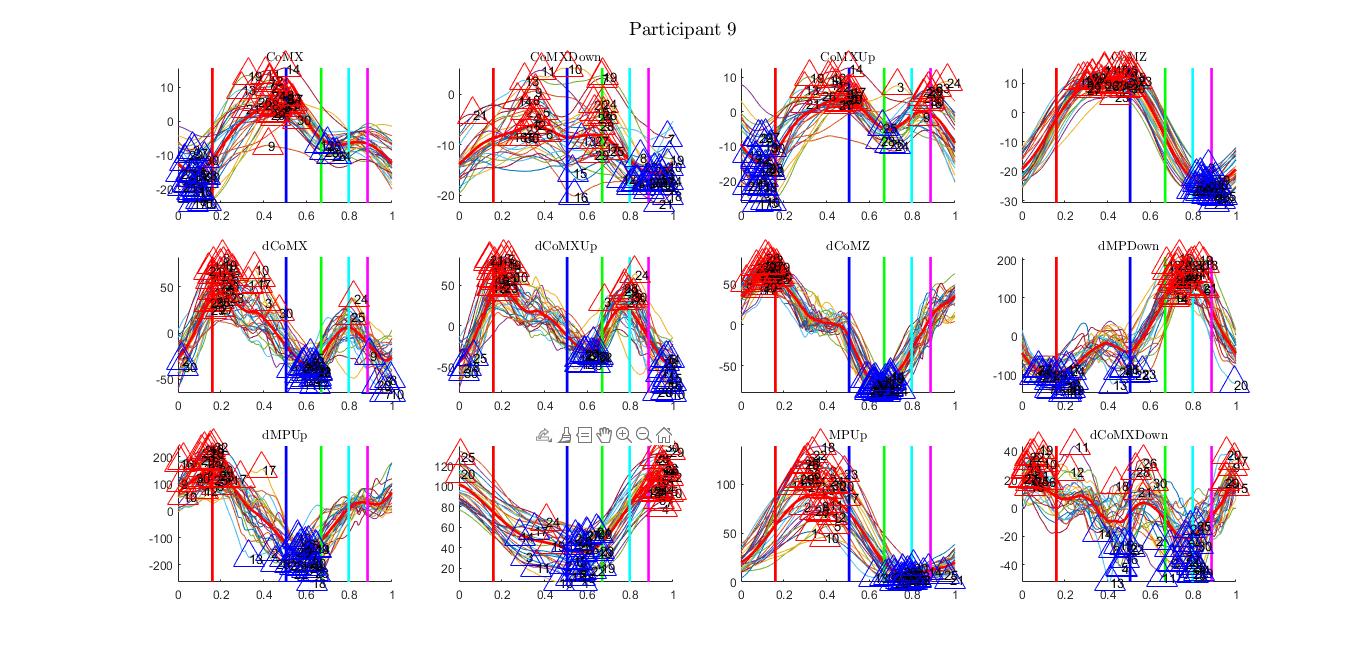

Supplement: Supplementary file 1 [file sensors-21-05632-s001.zip › Participant9.jpg]
